# Insight into the Burden of Malignant Respiratory Tumors and their Relationship with Smoking Rates and Lead Contamination in Mexico

**DOI:** 10.3390/toxics10110708

**Published:** 2022-11-20

**Authors:** Oliver Mendoza-Cano, Efrén Murillo-Zamora, Ángeles Catalina Ochoa-Martínez, Valeria Argentina Mendoza-Olivo, Mónica Ríos-Silva, Xóchitl Trujillo, Miguel Huerta, Jaime Alberto Bricio-Barrios, Verónica Benites-Godínez, Irma González-Curiel, Rebeca Yasmín Pérez-Rodríguez, Nadia Azenet Pelallo-Martínez, Agustín Lugo-Radillo

**Affiliations:** 1 Facultad de Ingeniería Civil, Universidad de Colima, km. 9 Carretera Colima-Coquimatlán, Coquimatlán C.P. 28400, Colima, Mexico; 2 Departamento de Epidemiología, Unidad de Medicina Familiar No. 19, Instituto Mexicano del Seguro Social, Av. Javier Mina 301, Col. Centro, Colima C.P. 28000, Colima, Mexico; 3 Facultad de Medicina, Universidad de Colima, Av. Universidad 333, Col. Las Víboras, Colima C.P. 28040, Colima, Mexico; 4 Laboratorio de Toxicología Molecular, Centro de Investigación Aplicada en Ambiente y Salud (CIAAS), Coordinación Para La Innovación y Aplicación de La Ciencia y La Tecnología (CIACyT), Universidad Autónoma de San Luis Potosí, Av. Sierra Leona 550, Col. Lomas Segunda Sección, San Luis Potosí C.P. 78210, San Luis Potosí, Mexico; 5 Facultad de Medicina, Universidad Autónoma de San Luis Potosí, Av. Venustiano Carranza 2405, Col. Lomas los Filtros, San Luis Potosí C.P. 78210, San Luis Potosí, Mexico; 6 Facultad de Ciencias Químicas, Universidad de Colima, km. 9 Carretera Colima-Coquimatlán, Coquimatlán C.P. 28400, Colima, Mexico; 7 Centro Universitario de Investigaciones Biomédicas, Universidad de Colima—CONACyT, Av. 25 de Julio 965, Col. Villas San Sebastián, Colima C.P. 28045, Colima, Mexico; 8 Centro Universitario de Investigaciones Biomédicas, Universidad de Colima, Av. 25 de Julio 965, Col. Villas San Sebastián, Colima C.P. 28045, Colima, Mexico; 9 Coordinación de Educación en Salud, Instituto Mexicano del Seguro Social, Calzada del Ejercito Nacional 14, Col. Fray Junípero Serra, Tepic C.P. 63160, Nayarit, Mexico; 10 Unidad Académica de Medicina, Universidad Autónoma de Nayarit, Ciudad de la Cultura Amado Nervo, Tepic C.P. 631555, Nayarit, Mexico; 11 Unidad Académica de Ciencias Químicas, Universidad Autónoma de Zacatecas, Campus UAZ, Siglo XXI. Carr. Zacatecas-Guadalajara Km. 6., Zacatecas C.P. 98160, Zacatecas, Mexico; 12 Department of Chemistry, DCNE, University of Guanajuato, Campus Guanajuato, Guanajuato C.P. 36700, Guanajuato, Mexico; 13 CONACYT—Faculty of Medicine and Surgery, Universidad Autónoma Benito Juárez de Oaxaca, Oaxaca C.P. 68020, Oaxaca, Mexico

**Keywords:** lead, environmental pollutants, global burden of disease, lung, neoplasms

## Abstract

We aimed to report the results from the Global Burden of Disease Study 2019 related to respiratory malignant tumors (tracheal, bronchial, and lung) in Mexico. We also evaluated the relationship between the burden of these neoplasms and the proportion of daily smokers and total lead emissions in 2019. A cross-sectional analysis of ecological data was performed. The burden of these tumors was 152,189 disability-adjusted life-years (DALYs), and years of life lost (YLL) contributed to 99% of them. The highest DALYs rates (per 100,000) were observed in the states of Sinaloa, Chihuahua, Baja California Sur, Sonora, and Nayarit. We documented a linear relationship between the DALYs rates and the prevalence of daily smokers (*β* = 8.50, 95% CI 1.58–15.38) and the total lead emissions (tons/year: *β* = 4.04, 95% CI 0.07–8.01). If later replicated, our study would provide insight into the major relevance of regulating tobacco use and the activities associated with the production of lead dust and other hazardous contaminants.

## 1. Introduction

In Mexico, as in the rest of the world, respiratory malignant tumors (tracheal, bronchial, and lung) are the leading cause of cancer-related deaths [1,2], and increasing trends in mortality rates have been documented [3]. Factors determining the latter are numerous and published data that suggest that nearly three out of four respiratory malignant tumors are attributable to potentially modifiable risk factors, including smoking [4].

Moreover, and since the corresponding population-attributable fraction of respiratory cancers is nearly two-thirds, tobacco use is the leading risk factor for these neoplasms [5]. Fine particulate air pollution may also play a role in the pathogenesis of these tumors [6]. Lead is a metal that can be found in fine particulate air pollution and is an established carcinogen in experimental animals [7,8]. The administration of inorganic lead to rats and mice via different routes resulted in the development of renal tumors, gliomas, and/or lung adenomas. In humans, this metal is classified as a possible carcinogen [9]. The results of observational epidemiologic studies investigating the association of lead exposure with cancer are inconsistent and vary according to the type of cancers reported [10,11,12].

In Mexico, the Pollutant Emissions and Transfer Register (RETC, its Spanish acronym), derived from the commitments acquired as a result of the signing of the Free Trade Agreement with the countries of North America (NAFTA), contains data on the substances that are emitted into the air, water, soil, and subsoil or are transferred in discharges to the sewage system and hazardous waste. Lead is one of the 200 substances included in this information system [13]. Data regarding tobacco exposure by the state of residence are also available in Mexico. These data surge from a probabilistic survey called the National Survey of Drug, Alcohol, and Tobacco Consumption (ENCODAT, its Spanish acronym).

In this article, we aimed to report the results from the Global Burden of Disease Study 2019 (GBD 2019) related to respiratory malignant tumors (namely tracheal, bronchial, and lung). We also evaluated the relationship between the burden of these neoplasms, the prevalence of daily smokers, and the total lead emissions reported by the RETC.

## 2. Methods

We performed a cross-sectional analysis that included three publicly available global and regional (Mexico) datasets. Data regarding the burden of tracheal (International Statistical Classification of Diseases and Related Health Problems, 10th revision [ICD-10], C33), bronchus, and lung (ICD-10 C34) cancer in the 32 states of Mexico were obtained from the Global Burden of Disease Study 2019 (GBD 2019) (https://vizhub.healthdata.org/gbd-results/, accessed on 21 October 2022). Retrieved information included the gender-stratified incidence and mortality rates (per 100,000), as well as the DALYs as absolute numbers and rates (per 100,000).

Data regarding the exposure to tobacco, specifically the prevalence of daily smokers per state, were retrieved from the latest (2017) ENCODAT (https://encuestas.insp.mx/repositorio/encuestas/ENCODAT2016/descargas.php, accessed on 21 October 2022). The mean environmental exposure to lead dust (tons/year) was retrieved from the levels of emission and transfer of substances, by state, from the RETC of the Ministry of Environment and Natural Resources of Mexico, corresponding to 2019 http://sinat.semarnat.gob.mx/retc/retc/index.php, accessed on 21 October 2022). Data for 24 out of 32 states of Mexico were available in the previously cited repository. The states that lacked information were Aguascalientes, Baja California Sur, Chiapas, Mexico City, Michoacán de Ocampo, Morelos, Nayarit, and Quintana Roo.

Summary statistics were computed. We used Spearman’s rank correlation coefficients (rho) and regression coefficients (β) and 95% confidence intervals (CI) computed through linear regression models to assess the relationship between the burden of the analyzed respiratory malignant tumors and the evaluated exposures. Since we analyzed aggregated publicly available data entirely for academic purposes, the evaluation of the research protocol by an ethics committee was waived.

## 3. Results

**Figure 1:**
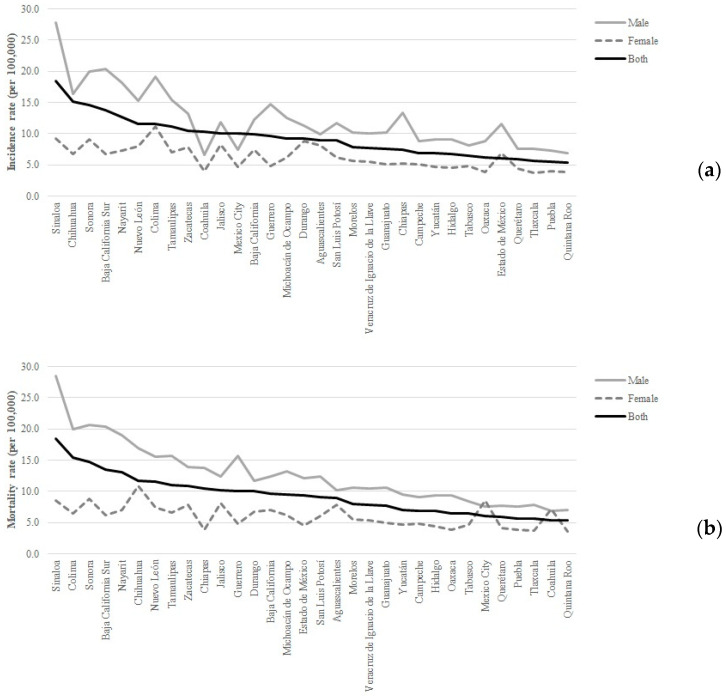
Sex-stratified incidence (**a**) and mortality (**b**) rates (per 100,000) due to tracheal, bronchus, and lung cancers in Mexico, 2019.

During 2019, the incidence rate (per 100,000) of respiratory-track malignant tumors in Mexico was 8.7 (95% CI 7.5–10.1) and it was higher among males (11.3 [95% CI 9.3–13.9] vs. 6.2 [95% CI 5.1–7.5]). The mortality rate (per 100,000) was 11.7 (95% CI 9.6–14.2) and 6.0 (95% CI 4.9–7.3) in males and females, respectively (overall: 8.8 [95% CI 7.5–10.2]). The state- and gender-stratified incidence and mortality rates are summarized in Figure 1.

As presented in Table 1, the total DALYs due to tracheal, bronchial, and lung cancers were 152,189 and the YLL contributed to 99% (n = 147,278) of the disease burden. The mean DALYs rate (per 100,000) was 197.3 and ranged from 113.1 (Tlaxcala) to 312.2 (Sinaloa). The rate in males almost doubled that observed in females (260.4 vs. 137.0 per 100,000).

**Figure 2:**
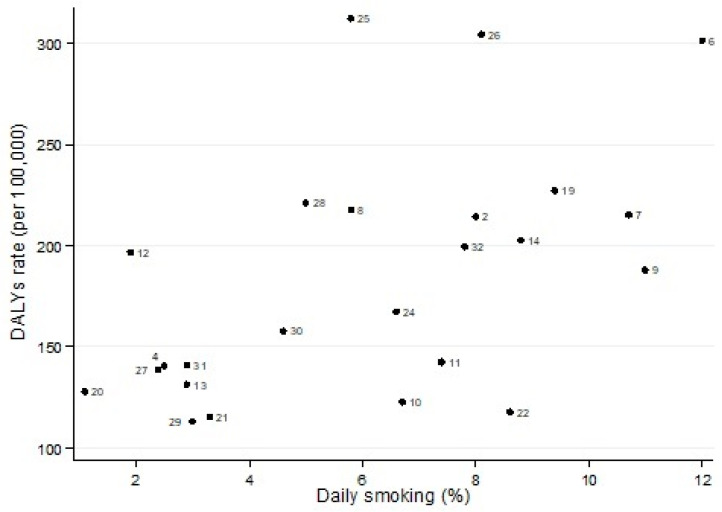
Proportion (%) of adult daily smokers and Disability-Adjusted Life-Years (DALYs) rate (per 100,000) due to tracheal, bronchus, and lung cancer in both genders, Mexico 2019. Note: (a) Spearman’s rank correlation coefficient (rho) was 0.56 (*p* = 0.001); (b) 2, Baja California; 4, Campeche; 6, Chihuahua; 7, Coahuila; 8, Colima; 9, Durango; 10, Estado de México; 11, Guanajuato; 12, Guerrero; 13, Hidalgo; 14, Jalisco; 17, Nuevo León; 20, Oaxaca; 21, Puebla; 22, Querétaro; 23, San Luis Potosí; 25, Sinaloa; 26, Sonora; 27, Tabasco; 28, Tamaulipas; 29, Tlaxcala; 30, Veracruz de Ignacio de la Llave; 31, Yucatán; 32, Zacatecas.

**Figure 3:**
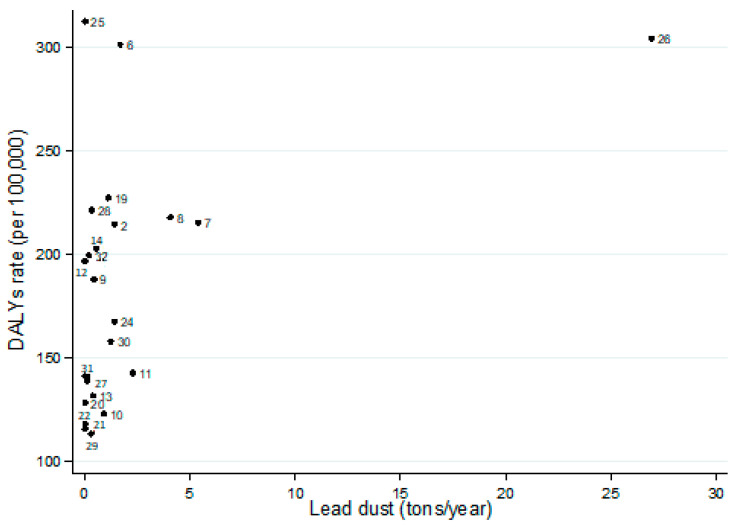
Mean environmental exposure to lead dust (tons/year) and Disability-Adjusted Life-Years (DALYs) rate (per 100,000) due to tracheal, bronchus, and lung cancer in both genders, Mexico 2019. Note: (a) Spearman’s rank correlation coefficient (rho) was: 0.48 (*p* = 0.017); (b) 2, Baja California; 4, Campeche; 6, Chihuahua; 7, Coahuila; 8, Colima; 9, Durango; 10, Estado de México; 11, Guanajuato; 12, Guerrero; 13, Hidalgo; 14, Jalisco; 17, Nuevo León; 20, Oaxaca; 21, Puebla; 22, Querétaro; 23, San Luis Potosí; 25, Sinaloa; 26, Sonora; 27, Tabasco; 28, Tamaulipas; 29, Tlaxcala; 30, Veracruz de Ignacio de la Llave; 31, Yucatán; 32, Zacatecas.

Considering the 24 states of Mexico where lead-related data were available, the median proportion of adult daily smokers was 6.2 and ranged (interquartile range) from 3.0 to 8.4. The states of Chihuahua (12.0%) and Oaxaca (1.1%) showed the highest and lowest prevalence, respectively (Figure 2). Spearman’s rank correlation coefficient (rho) was significant (rho = 0.56, p = 0.001).The median lead emissions were 0.4 tons/year and ranged (interquartile range) from 0.1 to 1.4 tons/year. The state of Sonora showed the highest emissions (27.0 tons/year). Figure 2 presents the relationship between DALYs rates and lead emissions in 24 states in which data regarding environmental exposure were available (Figure 3). The correlation coefficients were significant in the general analysis (rho = 0.48, p = 0.017). If the state of Sonora was excluded, the estimate was still significant (rho = 0.43, p = 0.040).

In a multiple regression analysis, the total lead emissions (tons/year: β = 4.04, 95% CI 0.07–8.01; p = 0.047) and the prevalence of daily smokers (β = 8.50, 95% CI 1.58–15.38; p = 0.018) were associated with the DALYs rates due to respiratory malignant tumors (adjusted R^2^ = 0.35). The raw data matrices are included in Supplementary Data S1.

## 4. Discussion

In our analysis, we observed a linear relationship between the burden of respiratory malignant tumors, the prevalence of daily smokers, and the normative environmental lead emissions. Our results, if later replicated, would be helpful to better understand the trends and variation of respiratory malignant tumors, which is critical to good health system planning and the development of epidemiologic vigilance and prevention programs.

In Mexico, policies focusing on the control of tobacco use have been implemented since 2007 when the taxes on these products increased from 54.2% to 58.9% of the final price [14]. These interventions, together with others under the World Health Organization’s Framework Convention on Tobacco Control, produced a decline in daily tobacco use in Mexico from 13.5% of the population to 7.7% from 2002 to 2009, but then remained virtually constant to date [15].

Mining and pesticide usage in Mexico will be addressed in this section to explain this potentially non-fortuity relationship between respiratory neoplasm and lead emissions. These two economic activities have high figures in two of the states, both located in Northwestern Mexico, which showed the highest disease burden: Sinaloa (312.2 DALYs per 100,000) and Sonora (304.3 DALYs per 100,000).

The state of Sonora generates 85% of the national production of copper and it is the main producer of gold. Approximately 60% of the extracted minerals are destined for export and, in addition, this state has the highest percentage of foreign investment in mining activities. This state of Mexico is also the main producer of gold and has the highest percentage of foreign investment in mining activities, with 60% of its production destined for export [16].

After the extraction and milling process, minerals of low economic value are placed on terraces within the mining site. The high suspension of fine dust particles (which may contain potentially toxic elements for humans and other living beings) in the air creates a hostile and toxic environment for workers and residents surrounding the mining site [17].

Currently, one of the biggest problems in the Mexican territory is the indiscriminate and uncontrolled use of pesticides. The current usage of these compounds is approximately 2 million tons per year [18]. However, there is currently a lack of knowledge on the amount and types of pesticides (active ingredients) that are applied in the fields.

The information available regarding the volume and types of pesticides applied annually in agricultural fields and the degree of organic contamination with toxic products in water bodies is practically non-existent. Until 2021, the Mexican states with the highest agricultural production were Jalisco, Veracruz, Oaxaca, Chihuahua, and Sinaloa. Their main products are grasses (Jalisco and Oaxaca), sugar cane (Veracruz and Jalisco), oranges (Veracruz), corn (Sinaloa and Chihuahua), alfalfa (Chihuahua), and tomatoes (Sinaloa) [19]. The areas with the highest use of pesticides in agriculture or for sanitary purposes in 2000 were Sinaloa, Chiapas, Veracruz, Jalisco, Nayarit, Colima, Sonora, Baja California, and Tamaulipas. These states accounted for approximately 70% of the consumption of pesticides [20].

The RETC, which served as the data source in our analysis, represents relevant progress in environmental health in Mexico. Access to environmental data must be guaranteed to increase awareness of environmental problems and promote effective public participation. This latter is fundamental to encouraging greater general understanding and support for environmental policies and enforcement [21].

In Mexico, as in many other countries of the world, public environmental information is lacking, and there is no strong legal framework to obligate private and public sectors to provide information to the Environmental ministry. There are also complex issues that affect law enforcement in general, ranging from Mexico’s relatively new democratic regime (which does not always make environmental matters a priority) to inappropriate practices in administrative and judicial structures and, more recently, severe budget cuts and budgetary constraints [22].

We also evaluated the prognostic value of the evaluated exposure (daily smoking prevalence and mean environmental exposure to lead dust) on respiratory cancers’ incidence rate (per 100,000) by using a multiple regression model. The prevalence of daily smokers (β = 44.12, 95% CI 3.10–85.14; p = 0.036) was associated with the incidence rates (adjusted R^2^ = 0.25), but the association of mean environmental exposure to lead was not significant (p = 0.142).

Finally, the potential limitations of our study must be cited. First, we used the mean environmental exposure to lead, and no biomarkers (i.e., blood levels in cancer patients) were not analyzed. Second, the statistical power of this study is limited by the relatively small cohort size and follow-up duration. Third, since we analyzed the general population, the levels of lead exposure are expected to be lower than those that would be found in occupationally exposed subjects. Fourth, the tumors of interest (tracheal, bronchial, and lung) are aggregated by the GBD study protocol, and we were unable to perform a stratified analysis. Fourth, our analysis is restricted to lead because, in other metals at RETC, the information was too limited to provide reliable estimates.

## 5. Conclusions

Our results suggest a linear relationship between the mean exposure to lead dust, the prevalence of daily smokers, and the burden of respiratory malignant tumors in Mexico. However, the limitations of our ecological approach must be considered in the interpretation of our findings. An observational study evaluating the analyzed association at an individual level would be highly useful from a public health and occupational perspective.

## Figures and Tables

**Table 1:** Burden of tracheal, bronchus, and lung cancer in the states of Mexico, 2019.

Rank/State		DALYs, *n*/Rate					
		All		Male		Female	
1	Sinaloa	11,746	312.2	8976	613.3	2770	186.1
2	Chihuahua	12,352	301.2	7899	451.9	4453	250.1
3	Sonora	9819	304.3	6912	460.7	2907	197.1
4	Baja California Sur	2737	256.6	2128	505.0	609	151.7
5	Nayarit	3481	238.2	2525	410.2	956	155.8
6	Nuevo León	13,404	227.2	9033	342.9	4371	165.7
7	Tamaulipas	8801	221.1	6118	353.5	2684	151.4
8	Colima	1998	217.6	1440	386.9	557	147.8
9	Coahuila	7393	215.0	4789	307.7	2604	165.7
10	Baja California	8986	214.1	5805	297.8	3181	166.6
11	Jalisco	18,429	202.7	10,957	272.4	7472	180.3
12	Zacatecas	3676	199.2	2300	291.8	1376	168.7
13	Mexico City	19,647	197.0	10,902	258.0	8745	190.6
14	Guerrero	8116	196.6	6016	335.7	2100	109.6
15	Durango	3710	187.9	2339	263.6	1371	151.4
16	Aguascalientes	2811	181.4	1551	228.5	1260	178.2
17	Michoacán de Ocampo	9520	175.5	6349	279.7	3171	132.3
18	San Luis Potosí	5564	167.4	3618	263.2	1946	134.8
19	Veracruz de Ignacio de la Llave	14,191	157.6	9003	228.5	5189	123.2
20	Morelos	3481	154.3	2214	232.2	1267	124.8
21	Guanajuato	10,509	142.5	6890	231.4	3619	113.6
22	Yucatán	3362	141.0	2208	205.0	1154	104.2
23	Campeche	1473	140.5	935	203.4	539	114.8
24	Tabasco	3782	138.8	2342	192.0	1440	113.8
25	Hidalgo	4607	131.4	2993	204.9	1614	103.2
26	Oaxaca	5741	128.0	3886	196.3	1855	85.8
27	Estado de México	24,605	122.8	14,842	175.7	9764	110.1
28	Querétaro	3007	117.8	1892	177.8	1115	99.6
29	Quintana Roo	2284	116.7	1497	173.3	787	93.5
30	Puebla	8287	115.3	5185	166.9	3101	91.8
31	Chiapas	7309	114.5	4443	154.1	2866	95.5
30	Tlaxcala	1718	113.1	1121	174.0	597	86.3
	National	152,189	197.3	99,657	260.4	52,532	137.0

Note: (1) The rate per 100,000 inhabitants is presented; (2) the states that lacked information regarding lead emissions were Aguascalientes, Baja California Sur, Chiapas, Mexico City, Michoacán de Ocampo, Morelos, Nayarit, and Quintana Roo. Abbreviations: DALYs, Disability-adjusted life years.

## Data Availability

Publicly available datasets were analyzed in this study. These data can be found here: https://vizhub.healthdata.org/gbd-results/ (accessed on 21 October 2022), http://sinat.semarnat.gob.mx/retc/retc/index.php (accessed on 21 October 2022), and https://encuestas.insp.mx/repositorio/encuestas/ENCODAT2016/descargas.php (accessed on 21 October 2022).

## Supplementary Materials

The following supporting information can be downloaded at: https://www.mdpi.com/article/10.3390/toxics10110708/s1, Data S1. DALYs rate due to malignant respiratory tumors, the prevalence of daily smokers, and lead contamination in Mexico in 2019.
