# Supplementary material for: Insight into the Burden of Malignant Respiratory Tumors and their Relationship with Smoking Rates and Lead Contamination in Mexico
Source: Toxics. 2022 Nov 20;10(11):708. doi: 10.3390/toxics10110708 (PMC9699460; doi:10.3390/toxics10110708)
Supplement: Supplementary file 1 [file toxics-10-00708-s001.zip › toxics-2025534-supplementary.pdf]

Supplementary data

Supplementary data S1. DALYs rate due to malignant respiratory tumors, the prevalence of daily smokers, and lead contamination, Mexico 2019

Location ID	DALYs rate (per 100,000)	State	Lead dust (tons/year)	Daily smokers (prevalence)
4643	181.4	Aguascalientes	-	0.102
4644	214.1	Baja California	1.423	0.080
4645	256.6	Baja California Sur	-	0.076
4646	140.5	Campeche	0.157	0.025
4647	215.0	Coahuila	5.404	0.107
4648	217.6	Colima	4.064	0.058
4649	114.5	Chiapas	-	0.013
4650	301.2	Chihuahua	1.678	0.120
4651	197.0	Mexico City	-	0.103
4652	187.9	Durango	0.427	0.110
4653	142.5	Guanajuato	2.284	0.074
4654	196.6	Guerrero	0.008	0.019
4655	131.4	Hidalgo	0.382	0.029
4656	202.7	Jalisco	0.539	0.088
4657	122.8	Estado de México	0.903	0.067
4658	175.5	Michoacán de Ocampo	-	0.066
4659	154.3	Morelos	-	0.042
4660	238.2	Nayarit	-	0.047
4661	227.2	Nuevo León	1.103	0.094
4662	128.0	Oaxaca	0.002	0.011
4663	115.3	Puebla	0.003	0.033
4664	117.8	Querétaro	0.027	0.086
4665	116.7	Quintana Roo	-	0.040
4666	167.4	San Luis Potosí	1.425	0.066
4667	312.2	Sinaloa	0.009	0.058
4668	304.3	Sonora	26.946	0.081
4669	138.8	Tabasco	0.109	0.024
4670	221.1	Tamaulipas	0.329	0.050
4671	113.1	Tlaxcala	0.289	0.030
4672	157.6	Veracruz de Ignacio de la Llave	1.247	0.046
4673	141.0	Yucatán	0.022	0.029
4674	199.2	Zacatecas	0.201	0.078

Abbreviation: **DALYs**, Disability-Adjusted Life Years.
Data sources: DALYs rate due to due to malignant respiratory tumors: Global Burden of Disease Study 2019 (GBD 2019) (https://vizhub.healthdata.org/gbd-results/). Prevalence of daily smokers: National Survey of Drug, Alcohol and Tobacco Consumption (ENCODAT) 2017 ((https://encuestas.insp.mx/repositorio/encuestas/ENCODAT2016/descargas.php). Mean environmental exposure to lead dust (tons/year): Pollutant Emissions and Transfer Register (RETC) (http://sinat.semarnat.gob.mx/retc/retc/index.php).
